# A Comparative Analysis of Ethnomedicinal Practices for Treating Gastrointestinal Disorders Used by Communities Living in Three National Parks (Korea)

**DOI:** 10.1155/2014/108037

**Published:** 2014-08-17

**Authors:** Hyun Kim, Mi-Jang Song, Heldenbrand Brian, Kyoungho Choi

**Affiliations:** ^1^School of Alternative Medicine and Health Science, Jeonju University, 303 Cheonjam-ro, Wansan-gu, Jeonju 560-759, Republic of Korea; ^2^Department of Integrated Bioresource Science, Graduate School of Jeonju University, 303 Cheonjam-ro, Wansan-gu, Jeonju 560-759, Republic of Korea; ^3^School of Liberal Arts, Jeonju University, 303 Cheonjam-ro, Wansan-gu, Jeonju 560-759, Republic of Korea; ^4^Department of Basic Medical Science, Jeonju University, 303 Cheonjam-ro, Wansan-gu, Jeonju 560-759, Republic of Korea

## Abstract

The purpose of this study is to comparatively analyze the ethnomedicinal practices on gastrointestinal disorders within communities in Jirisan National Park, Gayasan National Park, and Hallasan National Park of Korea. Data was collected through participant observations and indepth interviews with semistructured questionnaires. Methods for comparative analysis were accomplished using the informant consensus factor, fidelity level, and internetwork analysis. A total of 490 ethnomedicinal practices recorded from the communities were classified into 110 families, 176 genera, and 220 species that included plants, animals, fungi, and alga. The informant consensus factor values in the disorder categories were enteritis, and gastralgia (1.0), followed by indigestion (0.94), constipation (0.93), and abdominal pain and gastroenteric trouble (0.92). In terms of fidelity levels, 71 plant species showed fidelity levels of 100%. The internetwork analysis between disorders and all medicinal species are grouped in the center by the four categories of indigestion, diarrhea, abdominal pain, and gastroenteric trouble, respectively. Regarding the research method of this study, the comparative analysis methods will contribute to the availability of orally transmitted ethnomedicinal knowledge. Among the methods of analysis, the use of internetwork analysis as a tool for analysis in this study provides imperative internetwork maps between gastrointestinal disorders and medicinal species.

## 1. Introduction

After the agreement of the Nagoya Protocol, which has highlighted the importance of traditional knowledge of local communities, interest has grown stronger regarding ethnomedicinal knowledge in the world [1]. Ethnomedicinal knowledge plays an extremely vital role in the health care systems of developing countries and is utilized as an alternative for the treatment of disorders without side effects in developed countries [2]. Investigations regarding ethnomedicinal knowledge in local communities have often been conducted to the indigenous communities of Asia, Africa, and South America.

At present, studies on the ethnomedicinal practices of local communities to treat specific disorders have been accomplished, including liver disease [3, 4], birth-related diseases [5, 6], uremia [7], diabetes [8], psychiatric disorders [9], ophthalmology [10], skin disorders [11], stomach issues [11], veterinary medicine [12, 13], and other health conditions. However, research using INA on the ethnomedicinal practices to treat gastrointestinal disorders within local communities has yet to be accomplished.

Investigations for the ethnomedicinal practices of local communities to treat specific disorders in Korea have included respiratory diseases [14], digestive system disorders [15], and pain relief [16] for communities in North Jeolla Province.

National parks in Korea are areas designated to protect the representative ecosystem and the natural/cultural sceneries by the Ministry of Environment and are defined as natural areas of both land and sea. National parks are managed directly by the government and their purpose is to combine both a conservation and a sustainable use of the natural resources within the parks.

Designated as the first national park in 1967, Jirisan National Park spreads across one city and four counties and lies within three provinces. The total area of Jirisan National Park is 485 km^2^, which makes it the largest mountainous national park in Korea.

Hallasan National Park is located at the heart of Jeju Island, the largest and most beautiful island in Korea. Its total area is 1,849.18 km^2^ and is located at the southernmost tip of the nation.

Located in the deep inlands of southeastern Korea, Gayasan National Park spreads across one city and four counties and is located within two provinces. The total area of the park is 76.256 km^2^ and is known as the sacred site of Buddhism.

Accordingly, this research is the first attempt for comparing and analyzing ethnomedicinal practices to treat gastrointestinal disorders of communities in three national parks in Korea. However, up until now, a quantitative analysis for ethnomedicinal knowledge of local communities has relied solely on the consensus of its informants [17, 18] and the recorded fidelity levels [19–21].

These methods have limitations on the sufficient interpretation of ethnomedicinal knowledge as a complicated knowledge system embedded within the traditional ethnographical properties. Therefore, a deeper analysis of ethnomedicinal practices in treating specific disorders within the local communities is necessary for obtaining more specific details regarding the internetwork analysis (INA) between disorders and medicinal species.

This research suggests that the applications gained from utilizing the comparative INA for ethnomedicinal practices on gastrointestinal disorders within communities in three national parks will result in further research incorporating INA. The three study areas included in this study are Jirisan National Park (JNP), Gayasan National Park (GNP), and Hallasan National Park (HNP). These regions are included as typical inland and island areas of the southern region in Korea. Among the three national parks, the ethnomedicinal practices of the communities living within HNP were investigated in regard to both medicinal plants [22] and medicinal animals [23].

The results of this study can be utilized to develop functional foods, pharmafoods, and new ethnomedicinal practices for gastrointestinal disorders in these communities and other regions within Korea.

## 2. Research Area and Method

### 2.1. Natural and Social Environments of Research Area

**Figure 1:**
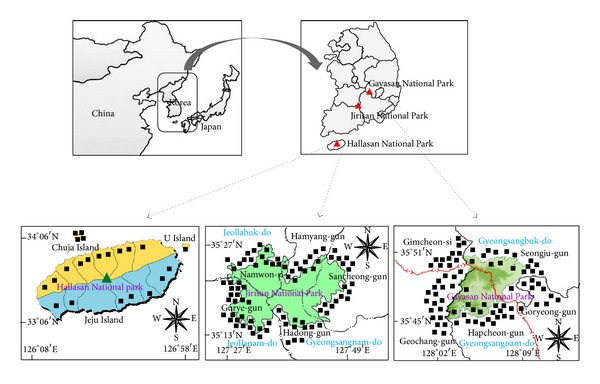
Investigation sites.

The study area consists of the southern region of the Korean peninsula and its many islands, which lie between 33° 06′N to 36° 09′N latitude and 125° 58′E to 128° 18′E longitude (Figure 1). The total population in 2012 of the study area was 1,161,002. The area measures approximately 2,410,434 km^2^ and includes five provinces, four cities, and eight counties in its administrative district [24]. The annual precipitation is around 1,200~2,300 mm in which the coastal area generally receives more rainfall than the inland regions. The annual average temperature of the inland regions is 13°C, while Jeju Island records 16.2°C [25]. The natural and social environments of the three national parks are summarized in Table 1.

### 2.2. Investigative Method

Field investigations were conducted from March 2009 to November 2012. Proper data was collected using participant observations and indepth interviews, as the informants also became investigators themselves through attending informal meetings, open and group discussions, and overt observations with semistructured questionnaires [21, 26].

The content of the semistructured questionnaires was composed of diverse information regarding medicinal species used to treat gastrointestinal disorders, including local names, used parts, methods of preparation, manufacturing and administration, dosage, and the usable duration regarding each curable formula [21, 27, 28].

All specimens were collected during their flowering or fruiting seasons and were organized utilizing the normal specimen manufacturing method [20, 27]. The voucher specimens were deposited for preservation in the herbarium of Jeonju University. The precise identification of species mentioned by the informants was performed in accordance with Lee [29], Lee [30], Ahn [31], Lee [32], and Park [33]. Scientific names were confirmed by the National Knowledge and Information System for Biological Species of Korea [34].

### 2.3. Quantitative Analysis

#### 2.3.1. Informant Consensus Factor (ICF)

The ICF was used to analyze the agreement degree of the informants' knowledge about each category of disorders [17, 18]. The ICF was calculated using the following formula:

(1) $$\begin{array}{c} \text{ICF}=\frac{\left(n_{ur}-n_{t}\right)}{\left(n_{ur}-1\right)}, \end{array}$$

where *n*_*ur*_ is the number of use reports of informants for a particular gastrointestinal disorder and *n*_*t*_ is the number of species used by all informants for a particular gastrointestinal disorder.

#### 2.3.2. Fidelity Level (FL)

The FL was employed to determine the most important species used for treating certain gastrointestinal disorders by the local practitioners and the elderly people living in the study area [19–21]. The FL was calculated using the following formula:

(2) $$\begin{array}{c} \text{FL}\left(\%\right)=N_{p}\times \frac{100}{N}, \end{array}$$

where *N*_*p*_ is the number of informants that mentioned the specific species used to treat certain disorders and *N* is the total number of the informants who utilized the species as medicine for treating any given disorder.

#### 2.3.3. Internetwork Analysis (INA)

Internetwork analysis does not focus on the independent characteristics of an individual within the community but considers the results of the interrelationship among each individual of a community. INA has been applied within communities for various ethnographical problems, including ethnogenesis [35] and obesity [36–38]. However, the INA had yet to be applied to ethnomedicinal knowledge, although it has been included in relation to its ethnographical properties.

Our research has newly applied this method in order to attain more internetwork information from the treatment of ethnomedicinal practices on gastrointestinal disorders within communities in Korea. The results of the INA of disorders and medicinal species were analyzed using UCINET (Ver. 6.460) and NetDraw (Ver. 2.125) software programs [39, 40].

## 3. Results and Discussion

### 3.1. Ethnographic Characteristics of the Region

The ethnomedicinal practices for gastrointestinal disorders were recorded by 507 informants (133 men and 374 women) at 185 sites (Figure 1). The average age of the informants was 76 years, with a range in age from 43 to 95, with residents living more than 30 years in the study area. The ethnographical characteristics of the communities are summarized in Table 2.

### 3.2. Analysis of Ethnomedicinal Practices

24 types of gastrointestinal disorders were treated by ethnomedicinal practices, which included abdominal pain, acute gastroenteritis, constipation, and other conditions (Table 3). The 24 types recorded in this study were similar to previous research, which classified 14 types of respiratory system diseases, 29 types of digestive system diseases, and 23 types of pain relief treatments [14, 16, 21]. Among them, 20 types of disorders were recorded in the communities living within JNP, followed by the 16 types of disorders within HNP, and the 11 types of disorders in GNP (Table 4).

A total of 490 ethnomedicinal practices recorded from the communities were classified into 110 families, 176 genera, and 220 species that included plants, animals, fungi, and alga (Table 4). Among these species, plants totaled 361 ethnomedicinal practices based on 142 species, while animals included 119 ethnomedicinal practices based on 71 species. Fungi recorded 9 ethnomedicinal practices based on six species while alga included one ethnomedicinal practice based on one species. These usage patterns were different from Korean traditional medicine, in which plants are used relatively much more than animals. Research confirms that communities have focused on the functional supplements from these ethnomedicinal practices rather than seeking after an actual cure for their gastrointestinal disorders.

The residents of these communities have applied the ethnomedicinal practices for gastroenteric trouble and indigestion more than any other disorder. Namely, the number of medicinal species and ethnomedicinal practices for gastroenteric trouble consisted of 94 species (42.7% of the total species) and 179 ethnomedicinal practices (36.5% of the total practices). Indigestion used 72 species (32.7% of the total species) and 131 ethnomedicinal practices (26.7% of the total practices) (Table 5).

Also, the number of informants who mentioned gastroenteric trouble and cases of indigestion occupied 28.9%, which totaled 30.0% of the whole, respectively (Table 5). As a result, the communities tended to use ethnomedicinal practices to care for their overall health instead of as a cure for a long-term condition.

For plants, 29 used parts were used in practice, while 14 used parts of animals and one used part of fungi and alga were used in treatment. Preparations of the plants consisted of 41 kinds, with 16 preparations for animals, six preparations for fungi, and one preparation for alga (Table 4). These usage patterns are similar to previous research for other diseases [14–16].

### 3.3. Quantitative Analysis

#### 3.3.1. Informant Consensus Factor (ICF)

The informant consensus factor ranges from 0 to 1, where the increasing values indicate a higher rate of informant consensus among the category of disorders.

The category with the highest degree of consensus from the informants were enteritis and gastralgia (1.0), followed by indigestion (0.94), constipation (0.93), abdominal pain and gastroenteric trouble (0.92), and gastric ulcers (0.91). The lowest degree of consensus was for gastroptosis, enterotoxin, hema feces, and other disorders (Table 6). These results denote that ethnomedicinal practices have been applied more often to minor health issues related to gastrointestinal disorders.

Generally, people suffering from serious gastrointestinal disorders have been treated in the hospital using conventional medicine or Korean traditional medicine. However, ethnomedicinal practices have been used to cure minor disorders.

Comparative consideration to results of the ICF among the three national parks and the agreement of consensus (ICF value, 1.00) from the informants in HNP obtained eight disorders, which include dysentery, gastralgia, gastric cancer, gastritis, hookworm, stomach cramps, stomach problems, and vomiting, while JNP and GNP depicted only enteritis and constipation, respectively.

These results confirm that the people of HNP have nearly the same ethnomedicinal knowledge for the treatment of gastrointestinal disorders because the communities have been isolated from other communities for many years.

#### 3.3.2. Fidelity Level (FL)

The FL is useful for identifying the informants' most preferred species in use for treating certain gastrointestinal disorders. This information reveals that the informants had a tendency to rely on one specific species for treating one specific disorder rather than for several different disorders. The FL values in this study varied from 1.0% to 100%.

Generally, a FL of 100% for a specific species indicates that all of the use-reports mentioned the same species for a specific treatment [41].

This study determined 71 species of plants with a FL of 100%, even without considering species that were mentioned more than two times (Table 3). Among them, plants with a FL of 100% in JNP totaled 52 species, followed by 40 species in GNP, and 23 species in HNP.

Disorders containing a higher number of species assessed to a FL of 100% were gastroenteric trouble (19 species) and cases of indigestion (22 species).

Special attention was given to important species (N, Np) with a FL above 100%, regarding the viewpoint of the number of times mentioned and the consensus level for the specific disorders, which include* Spiraea prunifolia* f.* simpliciflora* Nakai (224, 224),* Impatiens balsamina* L. as plants and* Acetes japonicus* Kishinouye (17, 17) as an animal cure for indigestion,* Xanthium strumarium* L. and* Petasites japonicas* (Siebold and Zucc.) Maxim. as plants used for curing gastroenteric trouble,* Zinnia violacea* Cav. and* Platycarya strobilacea* Siebold and Zucc. as plants used in treating abdominal pain, and* Viola verecunda* A. Gray as a plant used in treating dysentery (Table 3).

Through further study, these species possess a much higher potential in being used in the development of new functional supplements for treating specific gastrointestinal disorders.

#### 3.3.3. INA between Gastrointestinal Disorders and Medicinal Species

INA has originally analyzed social phenomenon and trends through the internetwork of components [42]. Our research has attempted to analyze the interrelationship between gastrointestinal disorders and the medicinal species recorded in the communities.

**Figure 2:**
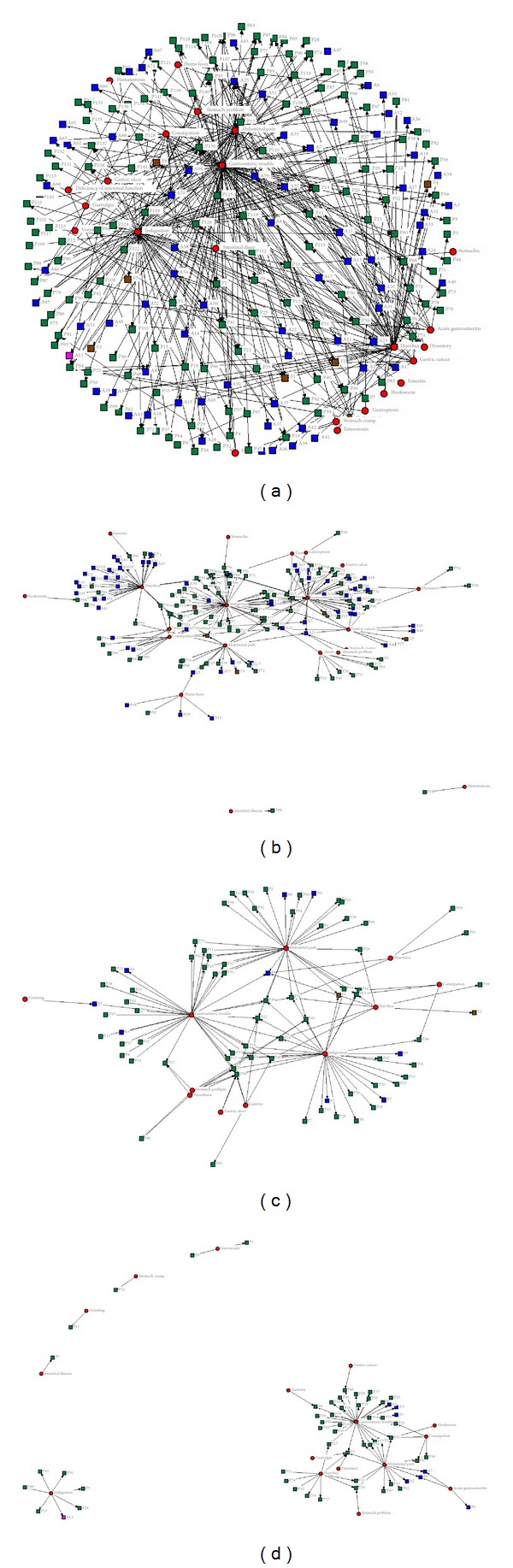
Internetwork analysis (INA) of three national parks ((a) total, (b) JNP, (c) GNP, and (d) HNP). ^∗^Abbreviation form of each is explained in Table 3: A (animal), P (plant), F (fungi), AL (algae), □ (species), and ◯ (disorders).

Considering Figure 2 about the internetwork between disorders and the medicinal species within all communities of this study, all medicinal species are grouped in the center for indigestion, diarrhea, abdominal pain, and gastroenteric trouble (Figure 2(a)), respectively. This distribution pattern is similar to the results of JNP and GNP. However, in case of HNP, indigestion is separated from the main disorders groups. This difference caused that the communities of HNP have been separated from the land communities for a long period of time.

In regard to the INA distribution map for JNP, the locations for the disorders of hema feces, intestinal disease, and hematemesis were fairly distinct from the four main disorders groups. Also, the cure for enteritis, hookworm, intestinal disease, stomach cramp, and stomachic is applied for only one medicinal species (Figure 2(b)).

In the case of GNP, gastritis, gastric ulcers, heartburn, and stomach problems were located as a distinct group separated from the four main disorder groups. Because this group consisted of minor stomach ailments having similar inclination,* Zanthoxylum piperitum* (L.) DC.,* Potentilla chinensis* Ser.,* Euonymus alatus* (Thunb.) Siebold,* Atractylodes ovate* (Thunb.) DC., and* Ulmus davidiana* var.* japonica* (Rehder) Nakai worked as possible cures as they possessed a high possibility in containing the same components for treatment (Figure 2(c)).

Within HNP, indigestion, intestinal disease, vomiting, stomach cramps, and enterotoxin were individually distinct from the three main disorder groups. This distribution pattern suggests that the application width of medicinal species to treat each disorder is limited for treating each disorder relative to the other communities (Figure 2(d)).

## 4. Conclusion

This research is the first study in the world to analyze and compare the ethnomedicinal practices of communities for treating gastrointestinal disorders. As the research method of this study, comparative quantitative analysis will contribute to the availability of orally transmitted ethnomedicinal knowledge. Additionally, the results of this study are confirmed due to the results obtained through investigation by 507 informants within the 185 research sites.

From this research, the recording of 490 ethnomedicinal practices being applied to the use of 220 medicinal species to treat 24 gastrointestinal disorders was extremely valuable. Particularly, the present usage of various medicinal species displays evidence as to which ethnomedicinal practices are continuously transmitted within the communities. However, this present situation is not sustainable because the communities of these study areas consist of an aging society. It has become necessary for appropriate measures to be taken to conserve these ethnomedicinal practices.

Our research suggests that treatment for gastroenteric trouble and indigestion among the gastrointestinal disorders uses ethnomedicinal practices more than any other type of treatment, as the communities used 75.5% of all medicinal species for treating these two diseases, 63.3% of the total number of all ethnomedicinal practices, and mentioned by 58.9% of all informants. Also, these two disorders contained the highest numbers of medicinal species within a FL of 100%. Through further study, the ethnomedicinal practices for these conditions possess a much higher potential in being used in the development of new practices.

According to the number of medicinal species applied to ethnomedicinal practices and the number of disorders treated by these ethnomedicinal practices, the numbers of JNP were much higher than the other two national parks. It is inferred that the region of JNP was the original center of Korean traditional medicine.

On the other hand, the communities of HNP depict a higher degree of agreement in the consensus to ethnomedicinal practices. This data explains that the communities of HNP, as island people, were limited in their movement to other regions and strictly collected large amounts of independent ethnomedicinal knowledge, only sharing within their own communities, which was distinct from the inland communities.

These trends were confirmed by the results of the INA as the internetwork maps of JNP and GNP were similar, while the map of HNP was moderately different. These results are reflected by the three-dimensional patterns of the ethnomedicinal knowledge held within the communities of each national park.

More specifically, the use of INA as a tool of quantitative analysis in this study provides valuable internetwork maps between gastrointestinal disorders and medicinal species.

These maps are important data to understand the specific interrelationships between disease and ethnomedicinal practices in the intra- and intercommunities.

The authors believe that INA is a useful new tool for providing various interpretations to ethnomedicinal knowledge in the intra- and intercommunities. This study provides confidence in that the useful value of INA will extend beyond the existing understanding of ethnomedicinal knowledge for the future research of ethnomedicinal knowledge.

## Figures and Tables

**Table 1:** Natural and social environments of three national parks.

Environment	JNP	GNP	HNP
Location	35°13′N~33°27′N 127°27′E~127°49′E	35°45′N~35°49′N 128°02′E~128°09′E	33°06′N~34°00′N 126°08′E~126°58′E
Administrative district	Three provinces, one city, and four counties	Two provinces, one city, and four counties	One province and two cities
Population (no.)	241,784	335,934	583,284
Area	485 km^2^	76.256 km^2^	1,849.18 km^2^
Annual precipitation	1,200~1,600 mm	1,200~1,600 mm	1,584~2,393 mm
Annual average temperature	12°C~14°C	11°C~13.2°C	15.6~16.9°C
Geographical characteristics	The center of the southern region of Korea	The southern region of Korea	The largest volcanic island in Korea
Climatic zone of vegetation	Between a warm temperate zone and a subarctic zone		Between a warm temperature zone to an alpine or arctic zone

∗JNP: Jirisan National Park, GNP: Gayasan National Park, HNP: Hallasan National Park.

**Table 2:** Ethnographical characteristics of three national parks.

Characteristics	JNP	GNP	HNP
Gender			
Male	67 (34.9%)	36 (15.6%)	31 (36.5%)
Female	125 (65.1%)	195 (84.4%)	54 (63.5%)
Average age	72.9 (44~95)	76.8 (52~93)	78.4 (43~94)
Educational attainment			
Never attended school	138 (71.9%)	165 (71.4%)	62 (72.9%)
Attended school	54 (28.1%)	66 (28.6%)	23 (27.1%)
Linguistics	The pronunciation between the eastern and western communities on the Jirisan axis depicts dissimilar intonations.		Numerous dialects different from the inland communities.
Food	The local communities in the eastern region of Jirisan widely used the seed powder of *Zanthoxylum piperitum* (L.) DC. and the leaves of the *Isodon japonicus *(Burm.) Hara, while local communities in the western region did not consume these foods.		Quite diverse from foods of the inland communities in regard to the recipe and ingredients.
Home economy	Men usually support their families financially.		Women traditionally support their families.

**Table 3:** Ethnomedicinal practices for treating gastrointestinal disorders recorded in three national parks.

Disorders	Classification	Family name	Scientific name	∗Abbreviation	∗∗Region	Korean name	Used part	Preparation	FL
	Animal	Anguillidae	*Anguilla japonica* Temminck and Schlegel	A6	JNP	Baemjangeo	Whole part	Simmer	50.00
		Apidae	*Apis cerana* Fabricius	A7	JNP	Jaeraekkulbeol	Honey	Raw	3.45
		Bombycidae	*Bombyx mori* L.	A12	GNP, JNP	Nuenabang	Larva, Pupa	Decoction and panbroiled	100.00
		Columbidae	*Streptopelia orientalis* Latham	A60	GNP	Metbidulgi	Meat	Simmer	33.33
		Congridae	*Conger myriaster* Brevoort	A18	HNP	Bungjangeo	Gallbladder	Raw	100.00
		Osmeridae	*Plecoglossus altivelis* Temminck and Schlegel	A45	JNP	Euneo	Whole part	Simmer	100.00
		Percichthyidae	*Lateolabrax japonicus* Cuvier and Valenciennes	A30	HNP	Nongeo	Gallbladder	Dried, dissolution, powder, and raw	85.71
		Phasianidae	*Gallus gallus domesticus* L.	A25	GNP, JNP	Dak	Whole part	Infusion	23.38
		Suidae	*Sus scrofa* L.	A62	HNP	Dwaeji	Gallbladder	Raw and maceration	40.00
	Fungi	Ganodermataceae	*Ganoderma lucidum* (Curtis) P. Karst	F2	GNP	Yeongji	Whole part	Boiling	22.22
		Pleurotaceae	*Lentinula edodes* (Berk.) Sing.	F3	JNP	Pyogo	Whole part	Porridge	100.00
		Polyporaceae	*Fomes fomentarius * (L.) Fr.	F1	JNP	Malgupbeoseot	Whole part	Decoction	33.33
		Actinidiaceae	*Actinidia arguta* (Siebold and Zucc.) Planch. ex Miq.	P4	GNP	Darae	Stem	Infusion	66.67
		Anacardiaceae	*Rhus javanica* L.	P106	JNP	Bungnamu	Gallnut	Decoction	100.00
			*Rhus verniciflua* Stokes	P107	JNP	Onnamu	Bark, stem	Infusion	11.43
		Araliaceae	*Aralia cordata* var. *continentalis* (Kitag.) Y.C.Chu	P14	GNP	Dokhwal	Root	Infusion	30.00
			*Eleutherococcus sessiliflorus* (Rupr. and Maxim.) S.Y.Hu	P42	JNP	Ogalpinamu	Stem	Decoction and infusion	100.00
		Asteraceae	*Artemisia princeps* Pamp.	P17	GNP, HNP, JNP	Ssuk	Aerial part, leaf, whole part, Young leaf	Infusion, juice, and tea	51.13
			*Cirsium japonicum* var. *maackii *(Maxim.) Matsum.	P28	GNP	Eonggeongkwi	Root	Juice	40.00
			*Zinnia violacea* Cav.	P141	JNP	Baegilhong	Stem	Infusion	100.00
		Brassicaceae	*Raphanus sativus* L.	P102	JNP	Mu	Root	Boiling and dried	100.00
		Campanulaceae	*Platycodon grandiflorum* (Jacq.) A.DC.	P86	GNP	Doraji	Root	Infusion	100.00
		Euphorbiaceae	*Ricinus communis* L.	P109	HNP	Pimaja	Seed	Oil	1.10
		Fabaceae	*Glycine max* (L.) Merr.	P50	JNP	Kong	Seed	Fermentation and dissolution	15.29
			*Glycyrrhiza uralensis* Fisch.	P51	GNP	Gamcho	Root	Infusion	46.15
			*Pueraria lobata* (Willd.) Ohwi	P97	GNP	Chik	Flower, root	Dried, decoction, grain syrup, and infusion	25.76
			*Sophora flavescens* Solander ex Aiton	P122	GNP	Gosam	Root	Maceration	6.25
Abdominal pain		Gentianaceae	*Gentiana scabra* Bunge	P47	GNP	Yongdam	Root	A sweet drink made from fermented rice and infusion	96.00
		Geraniaceae	*Geranium sibiricum* L.	P48	JNP	Jwisonipul	Whole part	Decoction	100.00
			*Geranium thunbergii* Siebold and Zucc.	P49	GNP, HNP	Ijilpul	Leaf, whole part	Decoction and infusion	76.92
		Juglandaceae	*Juglans mandshurica* Maxim.	P58	JNP	Garaenamu	Fruit	Raw	100.00
			*Platycarya strobilacea* Siebold and Zucc.	P85	GNP	Gulpinamu	Stem	Infusion	100.00
		Lamiaceae	*Leonurus japonicus* Houtt.	P61	GNP, HNP, JNP	Ingmocho	Aerial part	Decoction, infusion, juice, pill, and taffy	88.71
			*Mentha piperascens* (Malinv.) Holmes	P69	HNP	Bakha	Leaf	Juice	100.00
			*Perilla frutescens *var. *japonica *(Hassk.) Hara	P77	GNP	Deulkkae	Seed	Mixed in honey	63.64
	Plant		*Salvia plebeia* R.Br.	P114	GNP	Baeamchajeugi	Whole part	Infusion	50.00
		Lardizabalaceae	*Akebia quinata* (Houtt.) Decne.	P8	GNP	Eureumdeonggul	Stem	Infusion	85.71
		Liliaceae	*Allium scorodorprasum* var. viviparum Regel	P11	HNP	Maneul	Bulb	Juice	42.86
		Meliaceae	*Melia azedarach* L.	P68	HNP	Meolguseulnamu	Fruit	Decoction	47.37
		Papaveraceae	*Papaver somniferum* L.	P74	GNP, JNP	Yanggwibi	Fruit, leaf, stem, whole part	Brewing, decoction, dissolution, dried, grain syrup, infusion, maceration, and raw	82.54
		Phytolaccaceae	*Phytolacca esculenta* VanHoutte	P80	GNP	Jarigong	Root	Infusion	29.41
		Plantaginaceae	*Plantago asiatica* L.	P84	GNP	Jilgyeongi	Root	Juice	6.90
		Poaceae	*Hordeum vulgare* var. *hexastichon *(L.) Asch.	P54	GNP	Bori	Malt, seed	Dissolution and steep	5.52
			*Oryza sativa* L.	P72	JNP	Byeo	Seed	Porridge	42.86
			*Oryza sativa* var. *terrestris* Makino	P73	HNP	Sandu	Seed	Porridge	15.38
			*Triticum aestivum* L.	P129	HNP	Mil	Seed	Pill	3.85
			*Zea mays* L.	P139	GNP	Oksusu	Style	Infusion	100.00
		Punicaceae	*Punica granatum* L.	P99	GNP, HNP	Seongnyunamu	Fruit	Extraction, infusion, and raw	50.00
		Ranunculaceae	*Pulsatilla koreana* (Yabe ex Nakai) Nakai ex Nakai	P98	GNP	Halmikkot	Root	A sweet drink made from fermented rice, grain syrup, and infusion	44.78
		Rosaceae	*Prunus davidiana* (Carriere) Franch.	P93	GNP	Sanboksanamu	Fruit	Extraction	100.00
			*Prunus mume *Siebold and Zucc.	P94	GNP, JNP	Maesillamu	Fruit	Extraction	40.30
			*Sanguisorba officinalis* L.	P115	HNP	Oipul	Root	Decoction	22.22
		Rubiaceae	*Gardenia jasminoides* Ellis	P45	JNP	Chijanamu	Fruit	Decoction	100.00
		Rutaceae	*Phellodendron amurense* Rupr.	P79	HNP	Hwangbyeongnamu	Bark	Brewing and decoction	7.69
			*Zanthoxylum schinifolium* Siebold and Zucc.	P138	GNP	Sanchonamu	Fruit	Oil	21.43
		Schisandraceae	*Schisandra chinensis* (Turcz.) Baill.	P117	GNP	Omija	Stem	Infusion	66.67
		Solanaceae	*Solanum nigrum* L.	P121	GNP	Kkamajung	Aerial part	Infusion	11.76
		Theaceae	*Camellia japonica* L.	P21	HNP	Dongbaengnamu	Fruit	Oil	40.00
		Vitaceae	*Vitis coignetiae* Pulliat ex Planch.	P135	GNP	Meoru	Stem	Infusion	100.00

	Animal	Paralichthyidae	*Paralichthys olivaceus* Temminck and Schlegel	A41	HNP	Neopchi	Gallbladder	Dried	100.00
		Percichthyidae	*Lateolabrax japonicus* Cuvier and Valenciennes	A30	HNP	Nongeo	Gallbladder	Dried	14.29
		Suidae	*Sus scrofa* L.	A62	HNP	Dwaeji	Gallbladder	Dried	20.00
		Cucurbitaceae	*Cucumis sativus* L.	P35	JNP	Oi	Leaf	Raw	100.00
		Fabaceae	*Glycine max* (L.) Merr.	P50	JNP	Kong	Seed	Fermentation and dissolution	8.24
Acute gastroenteritis		Juglandaceae	*Juglans regia* Dode	P59	JNP	Hodunamu	Nut	Roast	100.00
		Liliaceae	*Allium scorodoprasum* var. viviparum Regel	P11	JNP	Maneul	Bulb	Roast	35.71
	Plant	Menispermaceae	*Cocculus trilobus* (Thunb.) DC.	P34	JNP	Daengdaengideonggul	Stem	Infusion	12.20
		Plantaginaceae	*Plantago asiatica* L.	P84	JNP	Jilgyeongi	Root	Juice	13.79
		Punicaceae	*Punica granatum* L.	P99	JNP	Seongnyunamu	Fruit	Brewing, juice, and raw	50.00
		Ranunculaceae	*Aconitum pseudolaeve* Nakai	P3	JNP	Jinbeom	Leaf, root	Decoction	100.00

		Gryllotalpidae	*Gryllotalpa orientalis* Burmeister	A28	JNP	Ttanggangaji	Whole part	Powder	83.33
		Mantidae	*Tenodera angustipennis* Saussure	A64	JNP	Samagwi	Egg sac	Decoction	50.00
	Animal		*Tenodera aridifolia* Stoll	A65	JNP	Wangsamagwi	Egg sac	Decoction	50.00
		Vespidae	*Vespa analis parallela* Andre	A67	JNP	Jommalbeol	Hive, imago, larva	Brewing	81.82
			*Vespa crabro flavofasciata* Cameron	A68	JNP	Malbeol	Hive, imago, larva	Brewing	87.10
			*Vespa simillimasimillima* Smith	A70	JNP	Teolbomalbeol	Hive, imago, larva	Brewing	81.82
	Fungi	Polyporaceae	*Fomes fomentarius* (L.: Fr.) Fr.	F1	JNP	Malgupbeoseot	Whole part	Decoction	33.33
		Asteraceae	*Ainsliaea acerifolia* Sch.Bip.	P7	JNP	Danpungchwi	Leaf	Seasoned cooked vegetables	100.00
		Boraginaceae	*Lithospermum erythrorhizon* Siebold and Zucc.	P63	JNP	Jichi	Root	Powder	11.11
Constipation		Cactaceae	*Opuntia ficus-indica* var. *saboten* Makino	P71	HNP	Sonbadakseoninjang	Stem	Raw	33.33
		Ebenaceae	*Diospyros kaki* Thunb.	P41	JNP	Gamnamu	Fruit	Fermentation	2.40
		Euphorbiaceae	*Ricinus communis* L.	P109	GNP, HNP	Pimaja	Fruit, seed	Oil and panfried	43.53
		Liliaceae	*Polygonatum odoratum* var. *pluriflorum* (Miq.) Ohwi	P87	JNP	Dunggulle	Root	Tea	40.00
	Plant		*Smilax china* L.	P120	JNP	Cheongmiraedeonggul	Fruit	Brewing	100.00
		Meliaceae	*Melia azedarach* L.	P68	HNP	Meolguseulnamu	Root	Decoction	21.05
		Phytolaccaceae	*Phytolacca esculenta* VanHoutte	P80	GNP	Jarigong	Root	Raw	35.29
		Rosaceae	*Prunus tomentosa* Thunb.	P95	JNP	Aengdonamu	Fruit	Brewing	100.00
		Rutaceae	*Zanthoxylum schinifolium* Siebold and Zucc.	P138	GNP	Sanchonamu	Fruit	Oil	14.29
		Saururaceae	*Saururus chinensis* (Lour.) Baill.	P116	JNP	Sambaekcho	Leaf	Decoction	50.00
		Theaceae	*Camellia japonica* L.	P21	HNP	Dongbaengnamu	Fruit	Oil	40.00

Deficiency of intestinal function		Apidae	*Apis mellifera* L.	A8	JNP	Yangbongkkulbeol	Honey	Raw	10.00
	Animal	Vespidae	*Vespa analis parallela* Andre	A67	JNP	Jommalbeol	Hive, larva	Decoction and infusion	18.18
			*Vespa crabro flavofasciata* Cameron	A68	JNP	Malbeol	Hive, larva	Decoction and infusion	12.90
			*Vespa simillima simillima* Smith	A70	JNP	Teolbomalbeol	Hive	Decoction and infusion	18.18
	Plant	Asteraceae	*Artemisia princeps* Pamp.	P17	JNP	Ssuk	Leaf	Tea	0.32

		Acrididae	*Anapodisma beybienkoi *Rentz and Miller	A5	JNP	Palgongsanmitdeurimettugi	Whole part	Panbroiled and powder	100.00
			*Arcyptera coreana* Shiraki	A9	JNP	Chameorisapsari	Whole part	Panbroiled and powder	100.00
			*Chorthippus nakazimai* Furukawa	A17	JNP	Suyeomchireaemettugi	Whole part	Panbroiled and powder	100.00
			*Gastrimargus marmoratus* Thunberg	A26	JNP	Kongjungi	Whole part	Panbroiled and powder	100.00
			*Locusta migratoria* L.	A32	JNP	Pulmuchi	Whole part	Panbroiled and powder	100.00
			*Megaulacobothrus aethalinus* Zubowsky	A34	JNP	Cheongnalgaeaemettugi	Whole part	Panbroiled and powder	100.00
			*Mongolotettix japonicus* Bolivar	A36	JNP	Sapsari	Whole part	Panbroiled and powder	100.00
			*Ognevia longipennis* Shiraki	A38	JNP	Ginnalgaemitdeurimettugi	Whole part	Panbroiled and powder	100.00
			*Ognevia sergii* Ikonnikovi Rehn and Rehni	A39	JNP	Wonsanmitdeurimettugi	Whole part	Panbroiled and powder	100.00
			*Oxya japonica japonica* Thunberg	A40	JNP	Byeomettugi	Whole part	Panbroiled and powder	100.00
			*Patanga japonica* Bolivar	A43	JNP	Gaksimettugi	Whole part	Panbroiled and powder	100.00
	Animal		*Shirakiacris shirakii* Bolivar	A58	JNP	Deunggeomeunmettugi	Whole part	Panbroiled and powder	100.00
			*Stethophyma magister* Rehn	A59	JNP	Kkeutgeomeunmettugi	Whole part	Panbroiled and powder	100.00
		Gryllidae	*Teleogryllus emma* Ohmachi and Matsumura	A63	JNP	Wanggwitturami	Whole part	Powder	100.00
		Megascolecidae	*Lumbricus rubellus* Hoffmeister	A33	JNP	Jireongi	Whole part	Simmer	100.00
		Phasianidae	*Gallus gallus domesticus* L.	A25	GNP	Dak	Egg	Infusion	6.49
		Pyrgomorphidae	*Atractomorpha lata* Motschulsky	A10	JNP	Seomseogumettugi	Whole part	Panbroiled and powder	100.00
		Ranidae	*Rana coreana* Okada	A48	JNP	Hanguksangaeguri	Whole part	Simmer	100.00
			*Rana huanrenensis *Fei, Ye, and Huang	A49	JNP	Gyegoksangaeguri	Whole part	Simmer	100.00
			*Rana nigromaculata* Okada	A50	JNP	Chamgaeguri	Whole part	Simmer	100.00
			*Rana temporaria dybowskii* Shannon	A51	JNP	Bukbangsangaeguri	Whole part	Simmer	100.00
		Suidae	*Sus scrofa* L.	A62	JNP	Dwaeji	Hide	Infusion	10.00
Diarrhea		Tubificidae	*Limnodrilus gotoi* Hatai	A31	JNP	Siljireongi	Whole part	Simmer	100.00
		Vespidae	*Vespa mandarinia* Cameron	A69	JNP	Jangsumalbeol	Hive, imago, and larva	Decoction	100.00
	Fungi	Ramariaceae	*Ramaria botrytis* (Pers.) Ricken	F4	GNP	Ssaribeoseot	Whole part	Infusion	100.00
		Anacardiaceae	*Rhus verniciflua* Stokes	P107	GNP	Onnamu	Stem	Burn, dissolution, powder,	2.86
		Araceae	*Pinellia ternata* (Thunb.) Breitenb.	P81	JNP	Banha	Corm	Decoction	50.00
		Asteraceae	*Artemisia princeps* Pamp.	P17	GNP, HNP, JNP	Ssuk	Aerial part, leaf, stem, root, and whole part	Decoction, extraction, infusion, juice, and moxibustion	20.90
			*Taraxacum platycarpum* Dahlst.	P125	JNP	Mindeulle	Whole part	Juice	14.29
		Dioscoreaceae	*Dioscorea batatas* Decne.	P39	HNP	Ma	Root	Infusion, raw	6.67
		Ebenaceae	*Diospyros kaki* Thunb.	P41	GNP, HNP, JNP	Gamnamu	Fruit and peduncle	Decoction, dried persimmon, infusion, and raw	30.40
		Geraniaceae	*Geranium thunbergii* Siebold and Zucc.	P49	HNP	Ijilpul	Whole part	Decoction	15.38
		Lamiaceae	*Leonurus japonicus* Houtt.	P61	JNP	Ingmocho	Aerial part	Infusion	3.23
		Liliaceae	*Allium fistulosum* L.	P9	JNP	Pa	Root	Infusion	100.00
			*Allium tuberosum* Rottler ex Spreng.	P12	JNP	Buchu	Whole part	Infusion	100.00
		Malvaceae	*Hibiscus hamabo* Siebold and Zucc.	P53	HNP	Hwanggeun	Root	Decoction	100.00
		Papaveraceae	*Papaver somniferum* L.	P74	GNP, JNP	Yanggwibi	Fruit, stem, and whole part	Decoction and dissolution	16.67
	Plant	Pinaceae	*Pinus densiflora* Siebold and Zucc.	P82	JNP	Sonamu	Endodermis	Infusion	100.00
		Plantaginaceae	*Plantago asiatica* L.	P84	HNP	Jilgyeongi	Whole part	Decoction	6.90
		Poaceae	*Oryza sativa* var. *terrestis* Makino	P73	HNP	Sandu	Seed	Porridge	76.92
			*Triticum aestivum* L.	P129	JNP	Mil	Seed	dissolution	7.69
		Polygonaceae	*Rheum rhabarbarum* L.	P104	HNP	Daehwang	Root	Decoction	100.00
			*Rumex acetosa *L.	P113	HNP	Suyeong	Root	Decoction	100.00
		Portulacaceae	*Portulaca oleracea* L.	P90	GNP	Soebireum	Aerial part	Seasoned cooked vegetables, seasoned with condiments	100.00
		Ranunculaceae	*Clematis trichotoma* Nakai	P33	JNP	Halmimilmang	Root	Decoction	100.00
			*Thalictrum aquilegifolium* var. sibiricum Regel and Tiling	P126	JNP	Kkwonguidari	Leaf and stem	Decoction	50.00
		Rutaceae	*Zanthoxylum schinifolium* Siebold and Zucc.	P138	GNP	Sanchonamu	Fruit	Oil	17.86
		Violaceae	*Viola mandshurica* W. Becker	P131	JNP	Jebikkot	Whole part	Decoction	100.00

	Animal	Phasianidae	*Gallus gallus domesticus* L.	A25	GNP	Dak	Egg	Infusion	5.19
		Asteraceae	*Artemisia princeps* Pamp.	P17	GNP, HNP	Ssuk	Aerial part, leaf, and whole part	Infusion, juice, and moxibustion	6.11
		Fabaceae	*Glycyrrhiza uralensis* Fisch.	P51	JNP	Gamcho	Root	Decoction and tea	15.38
			*Pueraria lobata* (Willd.) Ohwi	P97	JNP	Chik	Root	Decoction	0.76
Dysentery	Plant	Geraniaceae	*Geranium thunbergii* Siebold and Zucc.	P49	JNP	Ijilpul	Leaf	Decoction	7.69
		Polygonaceae	*Rheum rhabarbarum* L.	P104	JNP	Daehwang	Root	Decoction and tea	100.00
		Rosaceae	*Sanguisorba officinalis* L.	P115	GNP	Oipul	Whole part	Infusion	66.67
		Violaceae	*Viola verecunda* A. Gray	P132	GNP	Kongjebikkot	Leaf	Seasoned cooked vegetables	100.00

Enteritis	Plant	Ranunculaceae	*Thalictrum aquilegifolium* var. *sibiricum* Regel and Tiling	P126	JNP	Kkwonguidari	Leaf, stem	Decoction	50.00

Enterotoxin	Plant	Campanulaceae	*Adenophora triphylla* var. *japonica* (Regel) H. Hara	P6	HNP	Jandae	Root	Warm up in a double boiler	100.00
		Cucurbitaceae	*Cucurbita moschata* Duchesne	P36	HNP	Hobak	Fruit	Warm up in a double boiler	9.09

Gastralgia	Plant	Asteraceae	*Artemisia princeps* Pamp.	P17	HNP	Ssuk	Young leaf	Juice	1.61

		Formicidae	*Formica yessensis* Forel	A24	JNP	Bulgaemi	Whole part	Decoction	100.00
		Hominidae	*Homo sapiens* L.	A29	JNP	Saram	Bone	Burn, pill, and powder	100.00
	Animal	Muridae	*Rattus norvegicus* Berkenhout	A52	JNP	Jipjwi	Young rat	Fermentation	100.00
		Phasianidae	*Gallus gallus domesticus* L.	A25	JNP	Dak	Whole part	Decoction	1.30
		Scolopendridae	*Scolopendra subspinipes mutilans* L. Koch	A54	JNP	Jine	Whole part	Decoction	100.00
Gastric cancer	Fungi	Tricholomataceae	*Tricholoma matsutake* (S. Ito. and Imai) Sing.	F6	JNP	Songi	Whole part	Infusion	100.00
	Plant	Asteraceae	*Atractylodes ovata* (Thunb.) DC.	P19	JNP	Sapju	Root	Decoction	1.52
		Fabaceae	*Glycyrrhiza uralensis* Fisch.	P51	JNP	Gamcho	Root	Decoction and roast	7.69
		Poaceae	Hordeum vulgare var. hexastichon (L.) Asch.	P54	JNP	Bori	Seed	Decoction and roast	0.69
		Rutaceae	*Citrus unshiu* S. Marcov.	P30	JNP	Gyul	Pericarp	Decoction	50.00
			*Poncirus trifoliata* Raf.	P88	JNP	Taengjanamu	Fruit	Decoction	33.33
		Ulmaceae	*Ulmus davidiana* var. *japonica* (Rehder) Nakai	P130	HNP, JNP	Neureumnamu	Rhizodermis and root bark	Decoction	3.09

Gastric ulcer	Plant	Asteraceae	*Atractylodes ovata* (Thunb.) DC.	P19	JNP	Sapju	Root	Decoction	1.52
		Poaceae	*Hordeum vulgare* var. *hexastichon* (L.) Asch.	P54	GNP, JNP	Bori	Malt, seed	A sweet drink made from fermented rice, powder, roast, and steam	2.07
		Ulmaceae	*Ulmus davidiana* var*. japonica* (Rehder) Nakai	P130	GNP, JNP	Neureumnamu	Bark	A sweet drink made from fermented rice, decoction, infusion, and tea	11.73

		Anacardiaceae	*Rhus verniciflua* Stokes	P107	GNP	Onnamu	Bark	Simmer	2.86
		Asteraceae	*Atractylodes ovata* (Thunb.) DC.	P19	GNP	Sapju	Root	A sweet drink made from fermented rice and powder	6.06
		Ericaceae	*Rhododendron mucronulatum* Turcz. var. *mucronulatum *	P105	JNP	Jindallae	Flower	Panfried	66.67
		Poaceae	*Hordeum vulgare* var. *hexastichon* (L.) Asch.	P54	GNP	Bori	Malt, seed	A sweet drink made from fermented rice	1.38
Gastritis	Plant	Ranunculaceae	*Clematis terniflora* var. *mandshurica* (Rupr.) Ohwi	P32	HNP	Euari	Root	Taffy	57.14
		Rosaceae	*Rosa multiflora* Thunb. var. *multiflora *	P111	JNP	Jjillekkot	Flower, fruit	Decoction, panfried	87.50
		Rutaceae	*Citrus unshiu* S. Marcov.	P30	JNP	Gyul	Pericarp	Decoction	25.00
			*Zanthoxylum piperitum* (L.) DC.	P137	GNP	Chopinamu	Fruit	Oil	57.15
		Ulmaceae	*Ulmus davidiana* var. *japonica* (Rehder) Nakai	P130	GNP	Neureumnamu	Bark	A sweet drink made from fermented rice	1.23

		Apidae	*Apis cerana* Fabricius	A7	GNP	Jaeraekkulbeol	Honey	Dissolution and raw	93.10
			*Apis mellifera* L.	A8	GNP, JNP	Yangbongkkulbeol	Hive, honey, larva, whole part	Decoction, dissolution, power, and raw	90.00
		Blattellidae	*Blattella germanica* L.	A11	JNP	Bakwi	Whole part	Panbroiled and powder	100.00
		Cervidae	*Capreolus capreolus* L.	A13	JNP	Noru	Bone	Simmer	100.00
			*Capreolus pygargus tianschanicus* Satunin	A14	HNP	Noru	Bone	Simmer	100.00
		Colubridae	*Dinodon rufozonatumrufozonatum* Cantor	A19	JNP	Neunggureongi	Whole part	Simmer	100.00
			*Elaphe dione* Pallas	A20	JNP	Nurukbaem	Whole part	Simmer	100.00
			*Elaphe rufodorsata* Cantor	A21	JNP	Mujachi	Whole part	Simmer	100.00
			*Elaphe schrenckii* Strauch	A22	JNP	Gureongi	Whole part	Simmer	100.00
			*Gloydius ussuriensis* Emelianov	A27	JNP	Soesalmosa	Whole part	Simmer	100.00
			*Rhabdophis tigrinus tigrinus* Boie	A53	JNP	Yuhyeolmogi	Whole part	Simmer	100.00
		Columbidae	*Streptopelia orientalis* Latham	A60	JNP	Metbidulgi	Whole part	Simmer	66.67
		Cyprinidae	*Carassius auratus* L.	A15	JNP	Bungeo	Whole part	Simmer	100.00
	Animal	Erinaceidae	*Erinaceus amurensis* Schrenk	A23	GNP	Goseumdochi	Whole part	Infusion	100.00
		Gryllotalpidae	*Gryllotalpa orientalis* Burmeister	A28	JNP	Ttanggangaji	Whole part	Powder	16.67
		Mytilidae	*Mytilus coruscus* Gould	A37	HNP	Honghap	Whole part	Decoction	100.00
		Phasianidae	*Gallus gallus domesticus* L.	A25	GNP, HNP, JNP	Dak	Dung, whole part	Infusion, panbroiled, steep	61.04
			*Phasianus colchicus* L.	A44	JNP	Kkwong	Whole part	Simmer	100.00
		Pleuroceridae	*Semisulcospira coreana* Von Martens	A55	JNP	Chamdaseulgi	Whole part	Juice, panbroiled, powder, and simmer	90.91
			*Semisulcospira forticosta* Von Martens	A56	JNP	Jureumdaseulgi	Whole part	Juice, panbroiled, powder, and simmer	91.67
			*Semisulcospira libertina* Gould	A57	JNP	Daseulgi	Whole part	Juice, panbroiled, powder, and simmer	91.67
		Sphingidae	*Agrius convolvuli* L.	A4	JNP	Bakgaksi	Whole part	Infusion, maceration, and powder	100.00
		Suidae	*Sus scrofa* L.	A61	JNP	Dwaeji	Gallbladder	Dried, mixed in liquor, pill, and simmer	30.00
		Vespidae	*Vespula flaviceps lewisii* Cameron	A71	JNP	Ttangbeol	Hive, larva	Brewing and decoction	100.00
			*Gloydius blomhoffii brevicaudus* Stejneger	A2	JNP	Salmosa	Whole part	Simmer	100.00
			*Gloydius saxatilis* Emelianov	A3	JNP	Kkachisalmosa	Whole part	Simmer	100.00
	Fungi	Ganodermataceae	*Ganoderma lucidum* (Curtis) P. Karst.	F2	JNP	Yeongji	Whole part	Infusion	11.11
		Aceraceae	*Acer pictum* subsp. *mono* (Maxim.) Ohashi	P1	JNP	Gorosoenamu	Sap	Raw	100.00
		Actinidiaceae	*Actinidia arguta* (Siebold and Zucc.) Planch. ex Miq.	P4	JNP	Darae	Stem	Decoction	33.33
			*Actinidia polygama* (Siebold and Zucc.) Planch. ex Maxim.	P5	JNP	Gaedarae	Stem	A sweet drink made from fermented rice	100.00
		Anacardiaceae	*Rhus verniciflua* Stokes	P107	GNP, HNP, JNP	Onnamu	Bark, resin, stem, young leaf	Decoction, dissolution, extraction, infusion, raw, and simmer	72.14
		Apocynaceae	*Trachelospermum asiaticum* (Siebold and Zucc.) Nakai var. *asiaticum *	P127	JNP	Masakjul	Leaf, stem	Decoction	100.00
		Araliaceae	*Aralia cordata* var*. continentalis* (Kitag.) Y.C.Chu	P14	GNP	Dokhwal	Root	Maceration, mixed in liquor	60.00
			*Kalopanax *septemlobus (Thunb.) Koidz.	P60	GNP	Eumnamu	Stem	A sweet drink made from fermented rice and brewing	93.33
		Asteraceae	*Artemisia capillaris* Thunb.	P16	GNP	Sacheolssuk	Whole part	A sweet drink made from fermented rice, pill, and taffy	80.00
			*Artemisia princeps* Pamp.	P17	GNP, HNP	Ssuk	Leaf, whole part	Decoction, infusion, juice, moxibustion, and powder	12.22
			*Atractylodes ovata* (Thunb.) DC.	P19	GNP, JNP	Sapju	Root	A sweet drink made from fermented rice, decoction, dried, dissolution, infusion, pill, and powder	68.94
			*Cirsium japonicum* var. *maacki*i (Maxim.) Matsum.	P28	GNP, HNP	Eonggeongkwi	Root	Decoction and juice	60.00
			*Petasites japonicus* (Siebold and Zucc.) Maxim.	P78	GNP	Meowi	Leaf and stem	Infusion, wrapped in leaves, seasoned cooked vegetables	100.00
			*Taraxacum platycarpum* Dahlst.	P125	HNP, JNP	Mindeulle	Aerial part and whole part	Decoction, infusion, and tea	85.71
			*Xanthium strumarium* L.	P136	GNP	Dokkomari	Whole part	A sweet drink made from fermented rice and brewing	100.00
		Cactaceae	*Opuntia ficus-indica* var. *saboten* Makino	P71	HNP	Sonbadakseoninjang	Stem	Raw	66.67
		Caprifoliaceae	*Lonicera japonica* Thunb.	P64	HNP	Indongdeonggul	Flower	Decoction	33.33
		Celastraceae	*Euonymus alatus* (Thunb.) Siebold	P43	GNP, JNP	Hwasallamu	Leaf and stem	Decoction, seasoned cooked vegetables	40.54
			*Euonymus hamiltonianus* Wall. var. *hamiltonianus *	P44	JNP	Chambitsallamu	Stem	Infusion	100.00
Gastroenteric trouble		Crassulaceae	*Sedum sarmentosum* Bunge	P118	GNP	Dollamul	Whole part	Watery plain kimchi	100.00
		Cucurbitaceae	*Cucurbita moschata* Duchesne	P36	GNP	Hobak	Fruit	Infusion	90.91
			*Trichosanthes kirilowii* var. *japonica* Kitam.	P128	HNP	Noranghaneultari	Sap	Sap	100.00
		Dioscoreaceae	*Dioscorea batatas* Decne.	P39	GNP, JNP	Ma	Root	Decoction, maceration, oil, and raw	90.00
		Dioscoreaceae	*Dioscorea japonica* Thunb.	P40	GNP	Chamma	Root	Raw	100.00
		Ebenaceae	*Diospyros kaki* Thunb.	P41	GNP	Gamnamu	peduncle	Infusion	8.00
		Ericaceae	*Rhododendron mucronulatum* Turcz. var. *mucronulatum *	P105	JNP	Jindallae	Flower	Extraction	33.33
		Euphorbiaceae	*Ricinus communis* L.	P109	JNP	Pimaja	Seed	Oil	0.83
		Fabaceae	*Caragana sinica* (Buc'hoz) Rehder	P23	GNP	Goldamcho	Root	A sweet drink made from fermented rice	100.00
			*Glycine max* (L.) Merr.	P50	GNP, JNP	Kong	Seed	Dissolution and fermentation	37.65
			*Glycyrrhiza uralensis* Fisch.	P51	GNP, HNP, JNP	Gamcho	Root	Decoction	30.77
			*Pueraria lobata* (Willd.) Ohwi	P97	GNP, HNP, JNP	Chik	Root	Boiled rice, brewing, decoction, infusion, juice, and maceration	46.21
			*Sophora flavescens* Solander ex Aiton	P122	GNP, HNP	Gosam	Fruit, root	Decoction, infusion, and raw	68.75
		Lamiaceae	*Leonurus japonicus* Houtt.	P61	HNP	Ingmocho	Aerial part	Decoction	6.45
	Plant		*Perilla frutescens* var. *japonica* (Hassk.) Hara	P77	GNP	Deulkkae	Seed	Seasoned cooked vegetables	36.36
			*Salvia plebeia* R.Br.	P114	GNP, JNP	Baeamchajeugi	Leaf, whole part	Decoction and infusion	50.00
		Lauraceae	*Machilus thunbergii* Siebold and Zucc.	P66	HNP	Hubangnamu	Bark	Decoction	75.00
		Liliaceae	*Allium scorodoprasum* var. *viviparum* Regel	P11	HNP	Maneul	Bulb	Decoction	21.43
			*Polygonatum odoratum* var. *pluriflorum* (Miq.) Ohwi	P87	HNP	Dunggulle	Root	Tea	40.00
		Meliaceae	*Melia azedarach* L.	P68	HNP	Meolguseulnamu	Root bark	Taffy	5.26
		Menispermaceae	*Cocculus trilobus* (Thunb.) DC.	P34	GNP	Daengdaengideonggul	Root, stem	Infusion and maceration	78.05
		Moraceae	*Morus bombycis* Koidz. var. *bombycis *	P70	JNP	Sanppongnamu	Root	Decoction and infusion	100.00
		Oleaceae	*Ligustrum obtusifolium* Siebold and Zucc.	P62	JNP	Jwittongnamu	Fruit	Decoction	100.00
		Orchidaceae	*Gastrodia elata* Blume	P46	JNP	Cheonma	Tuber	Porridge	100.00
		Papaveraceae	*Papaver somniferum* L.	P74	HNP	Yanggwibi	Latex	Extraction	0.79
		Pinaceae	*Pinus koraiensis* Siebold and Zucc.	P83	JNP	Jannamu	Seed	Raw	100.00
		Plantaginaceae	*Plantago asiatica* L.	P84	GNP, HNP	Jilgyeongi	Leaf, petiole, whole part	Decoction	72.41
		Poaceae	*Hordeum vulgare* var. *hexastichon *(L.) Asch.	P54	GNP, JNP	Bori	Malt, seed	A sweet drink made from fermented rice, pill, and taffy	33.79
			*Triticum aestivum* L.	P129	GNP	Mil	Seed	A sweet drink made from fermented rice and brewing	50.00
		Ranunculaceae	*Aconitum ciliare* DC.	P2	JNP	Notjeotgarangnamul	Root	Infusion and pill	14.29
			*Clematis florida* Thunb.	P31	JNP	Wiryeongseon	Root	Decoction	100.00
			*Clematis terniflora* var. *mandshurica* (Rupr.) Ohwi	P32	HNP	Euari	Root	Decoction	42.86
			*Pulsatilla koreana* (Yabe ex Nakai) Nakai ex Nakai	P98	GNP, JNP	Halmikkot	Root	A sweet drink made from fermented rice, grain syrup, and infusion	41.79
		Rhamnaceae	*Ziziphus jujuba* var*. inermis* (Bunge) Rehder	P142	GNP, HNP	Daechunamu	Fruit	Infusion, simmer	100.00
		Rosaceae	*Prunus mume* Siebold and Zucc.	P94	JNP	Maesillamu	Fruit	Extraction	2.99
			*Rosa davurica* Pall.	P110	JNP	Saengyeolgwinamu	Fruit	Brewing	100.00
			*Sanguisorba officinalis* L.	P115	HNP	Oipul	Root	Decoction	11.11
		Rutaceae	*Phellodendron amurense* Rupr.	P79	HNP	Hwangbyeongnamu	Inner layer of bark	Decoction	15.38
			*Zanthoxylum schinifolium* Siebold and Zucc.	P138	JNP	Sanchonamu	Seed	Oil	21.43
		Salicaceae	*Populus maximowiczii* A. Henry	P89	GNP	Hwangcheollamu	Endodermis	Infusion	100.00
		Saururaceae	*Houttuynia cordata* Thunb.	P55	HNP	Yangmomil	Whole part	Decoction	100.00
			*Saururus chinensis* (Lour.) Baill.	P116	HNP	Sambaekcho	Whole part	Infusion	50.00
		Schisandraceae	*Schisandra chinensis* (Turcz.) Baill.	P117	HNP	Omija	Fruit, root, stem	Brewing	33.33
		Scrophulariaceae	*Rehmannia glutinosa* (Gaertn.) Libosch. ex Steud.	P103	JNP	Jihwang	Root	Brewing	100.00
		Solanaceae	*Lycium chinense* Mill.	P65	GNP, HNP	Gugijanamu	Fruit	Decoction and tea	100.00
		Ulmaceae	*Ulmus davidiana* var. *japonica *(Rehder) Nakai	P130	GNP, HNP, JNP	Neureumnamu	Bark, leaf, endodermis, rhizodermis, root, stem	A sweet drink made from fermented rice, boiling, decoction, dried, infusion, powder, simmer, and tea	71.60
		Zingiberaceae	*Curcuma longa* L.	P38	JNP	Ulgeum	Root	Tea	100.00
			*Zingiber officinale* Roscoe	P140	HNP	Saenggang	Rhizome	Simmer	100.00

Gastroptosis	Plant	Asteraceae	*Atractylodes ovata* (Thunb.) DC.	P19	JNP	Sapju	Root	Decoction	0.76
		Gentianaceae	Gentiana scabra Bunge	P47	JNP	Yongdam	Root	Tea	4.00
		Poaceae	*Hordeum vulgare* var. *hexastichon* (L.) Asch.	P54	JNP	Bori	Seed	Powder, roast, and steam	0.69

Heartburn	Plant	Asteraceae	*Atractylodes ovata* (Thunb.) DC.	P19	GNP	Sapju	Root	Powder	2.27
		Celastraceae	*Euonymus alatus* (Thunb.) Siebold	P43	GNP	Hwasallamu	Leaf, stem	Decoction and seasoned cooked vegetables	29.73
		Rosaceae	*Potentilla chinensis* Ser.	P91	GNP	Ttakjikkot	Root	Infusion and raw	50.00
			*Prunus mume* Siebold and Zucc.	P94	GNP	Maesillamu	Fruit	Extraction	3.73
		Ulmaceae	*Ulmus davidiana* var*. japonica* (Rehder) Nakai	P130	GNP	Neureumnamu	Bark	Tea	1.85

Hema feces	Animal	Anguillidae	*Anguilla japonica* Temminck and Schlegel	A6	JNP	Baemjangeo	Whole part	Simmer	50.00
			*Cetonia pilifera* Motschulsky	A16	JNP	Kkonmuji	Larva	Brewing	100.00
			*Protaetia brevitarsis seulensis* Kolbe	A46	JNP	Huinjeombagikkonmuji	Larva	Brewing	100.00
			*Protaetia mandschuriensis* Schurhoff	A47	JNP	Manjujeombagikkonmuji	Larva	Brewing	100.00
	Plant	Rosaceae	*Rosa rugosa* Thunb. var. *rugosa *	P112	JNP	Haedanghwa	Leaf, root	Decoction	100.00

Hematemesis	Plant	Theaceae	*Camellia japonica* L.	P21	JNP	Dongbaengnamu	Fruit	Decoction	20.00

Hookworm	Plant	Araceae	*Pinellia ternata* (Thunb.) Breitenb.	P81	JNP	Banha	Corm	Decoction	50.00
		Meliaceae	*Melia azedarach* L.	P68	HNP	Meolguseulnamu	Fruit, leaf, root bark	Decoction, taffy	26.32

	Alga	Gelidiaceae	*Gelidium amansii* J. V. Lamour.	AL1	HNP	Umutgasari	Thallus	Decoction	100.00
	Animal	Cobitidae	*Misgurnus mizolepis* Günther	A35	GNP	Mikkuraji	Whole part	Raw	100.00
		Gadidae	*Theragra chalcogramma* Pallas	A66	JNP	Myeongtae	Whole part	Decoction	100.00
		Mantidae	*Tenodera angustipennis* Saussure	A64	JNP	Samagwi	Whole part	Powder	50.00
			*Tenodera sinensis* Saussure	A65	GNP, JNP	Wangsamagwi	Whole part	Infusion, powder	50.00
		Octopodidae	*Paroctopus dofleini* Wulker	A42	JNP	Muneo	Whole part	Simmer	100.00
		Pleuroceridae	*Semisulcospira coreana* Von Martens	A55	JNP	Chamdaseulgi	Whole part	Simmer	9.09
			*Semisulcospira forticosta* Von Martens	A56	JNP	Jureumdaseulgi	Whole part	Simmer	8.33
			*Semisulcospira libertina* Gould	A57	JNP	Daseulgi	Whole part	Simmer	8.33
		Sergestidae	*Acetes japonicus* Kishinouye	A1	GNP	Jeotsaeu	Whole part	Fermentation	100.00
	Fungi	Ganodermataceae	*Ganoderma lucidum* (Curtis) P. Karst.	F2	GNP	Yeongji	Whole part	Infusion	66.67
		Polyporaceae	*Fomes fomentarius* (L.: Fr.) Fr.	F1	JNP	Malgupbeoseot	Whole part	Decoction	33.33
		Thelephoraceae	*Sarcodon aspratus* (Berk.) S. Ito	F5	JNP	Neungi	Whole part	Decoction, seasoned cooked vegetables, soup	100.00
		Amaranthaceae	*Amaranthus mangostanus* L.	P13	JNP	Bireum	Leaf	Dried persimmon, infusion	100.00
		Anacardiaceae	*Rhus verniciflua* Stokes	P107	GNP, JNP	Onnamu	Stem	Infusion	10.71
		Araliaceae	*Aralia cordata* var. *continentalis *(Kitag.) Y. C. Chu	P14	JNP	Dokhwal	Root	Decoction, tea	10.00
			*Aralia elata* (Miq.) Seem.	P15	JNP	Dureumnamu	Root	Infusion	100.00
			*Kalopanax septemlobus* (Thunb.) Koidz.	P60	GNP	Eumnamu	Stem	Infusion	6.67
		Aristolochiaceae	*Asarum sieboldii* Miq.	P18	JNP	Jokdoripul	Root	Infusion, pill	100.00
		Aspleniaceae	*Pteridium aquilinum* var. *latiusculum* (Desv.) Underw. ex Hell.	P96	JNP	Gosari	Root	Decoction, infusion	100.00
		Asteraceae	*Artemisia capillaris* Thunb.	P16	GNP	Sacheolssuk	Leaf, stem	Decoction	20.00
			*Artemisia princeps* Pamp.	P17	GNP, JNP	Ssuk	Leaf, root, whole part	Juice	7.72
			*Atractylodes ovata* (Thunb.) DC.	P19	GNP, JNP	Sapju	Root	Brewing, decoction, infusion, panbroiled, pill, powder	18.18
			*Helianthus annuus* L.	P52	JNP	Haebaragi	Flower	Decoction	100.00
			*Inula helenium* L.	P57	JNP	Mokhyang	Root	Decoction	100.00
		Balsaminaceae	*Impatiens balsamina* L.	P56	GNP, JNP	Bongseonhwa	Flower, root, whole part	Infusion	100.00
		Betulaceae	*Betula costata* Trautv.	P20	GNP	Geojesunamu	Sap	Raw	100.00
		Bignoniaceae	*Catalpa ovata* G. Don	P26	GNP	Gaeodong	Stem	Infusion	100.00
		Boraginaceae	*Lithospermum erythrorhizon* Siebold and Zucc.	P63	GNP	Jichi	Root	Panbroiled, steep	88.89
		Caprifoliaceae	*Lonicera japonica* Thunb.	P64	JNP	Indongdeonggul	Stem	Infusion	66.67
		Cyperaceae	*Carex curta* Gooden.	P24	JNP	Sansacho	Fruit	Decoction	100.00
		Dioscoreaceae	*Dioscorea batatas* Decne.	P39	JNP	Ma	Root	Decoction	3.33
		Ebenaceae	*Diospyros kaki* Thunb.	P41	GNP, JNP	Gamnamu	Fruit, peduncle	Dried persimmon, infusion, raw	59.20
Indigestion		Euphorbiaceae	*Ricinus communis* L.	P109	GNP, JNP	Pimaja	Fruit, seed	Oil, panfried	54.55
		Fabaceae	*Glycine max* (L.) Merr.	P50	JNP	Kong	Seed	Fermentation, dissolution	38.82
			*Pueraria lobata* (Willd.) Ohwi	P97	GNP	Chik	Root	Clear soup with flour dumplings, juice, tea	27.27
			*Rhynchosia volubilis* Lour.	P108	JNP	Jwinunikong	Seed	Decoction	100.00
			*Sophora flavescens* Solander ex Aiton	P122	JNP	Gosam	Root	Decoction, infusion, juice, maceration, panbroiled	25.00
		Fagaceae	*Castanea crenata* Siebold and Zucc.	P25	GNP, JNP	Bamnamu	Bark, nut	Infusion, tea	100.00
		Lamiaceae	*Leonurus japonicus* Houtt.	P61	JNP	Ingmocho	Aerial part	Juice	1.61
			*Perilla frutescens* var. *acuta* Kudo	P76	JNP	Soyeop	Leaf	Decoction	100.00
	Plant	Lardizabalaceae	*Akebia quinata* (Houtt.) Decne.	P8	JNP	Eureumdeonggul	Stem	Infusion	14.29
		Lauraceae	*Machilus thunbergii* Siebold and Zucc.	P66	JNP	Hubangnamu	Bark	Decoction	25.00
		Liliaceae	*Allium microdictyon* Prokh.	P10	JNP	Sanmaneul	Root	Decoction	50.00
			*Polygonatum odoratum* var. *pluriflorum* (Miq.) Ohwi	P87	JNP	Dunggulle	Root	Tea	20.00
		Loranthaceae	*Viscum album* var. *coloratum* (Kom.) Ohwi	P133	JNP	Gyeousari	Whole part	A sweet drink made from fermented rice	100.00
		Menispermaceae	*Cocculus trilobus* (Thunb.) DC.	P34	GNP	Daengdaengideonggul	Root	Juice	9.76
		Moraceae	*Cudrania tricuspidata* (Carr.) Bureau ex Lavallee	P37	JNP	Kkujippongnamu	Root	Decoction	100.00
		Phytolaccaceae	*Phytolacca esculenta* VanHoutte	P80	GNP, JNP	Jarigong	Root	A sweet drink made from fermented rice, infusion	35.29
		Poaceae	*Hordeum vulgare *var. *hexastichon* (L.) Asch.	P54	GNP, HNP, JNP	Bori	Malt, seed	A sweet drink made from fermented rice, brewing, decoction, dissolution, infusion, juice, maceration, steep, tea	55.86
			*Oryza sativa* L.	P72	GNP	Byeo	Stem	Infusion	57.14
			*Setaria italica* (L.) P. Beauv.	P119	HNP	Jo	Seed	A sweet drink made from fermented rice	100.00
			*Triticum aestivum* L.	P129	GNP	Mil	Seed	Brewing, clear soup with flour dumplings, extraction	38.46
		Ranunculaceae	*Aconitum ciliare* DC.	P2	JNP	Notjeotgarangnamul	Root	Decoction, infusion, pill	85.71
			*Pulsatilla koreana* (Yabe ex Nakai) Nakai ex Nakai	P98	JNP	Halmikkot	Root	Grain syrup	5.97
		Rosaceae	*Malus sieboldii* (Regel) Rehder	P67	JNP	Ageubaenamu	Fruit	Decoction, infusion	100.00
			*Prunus armeniaca* var. *ansu *Maxim.	P92	GNP, JNP	Salgunamu	Seed	Maceration, raw	100.00
			*Prunus mume* Siebold and Zucc.	P94	GNP, HNP, JNP	Maesillamu	Fruit	Brewing, dissolution, extraction	49.25
			*Pyrus pyrifolia* (Burm. f.) Nakai	P100	JNP	Dolbaenamu	Fruit	Brewing	100.00
			*Pyrus pyrifolia* var. *culta* (Makino) Nakai	P101	GNP	Baenamu	Fruit	Infusion	100.00
			*Rosa multiflora* Thunb. var. *multiflora *	P111	JNP	Jjillekkot	Fruit	Decoction	12.50
			*Spiraea prunifolia* f. *simpliciflora *Nakai	P124	GNP	Jopamnamu	Root, stem	Infusion	100.00
		Rutaceae	*Citrus unshiu* S. Marcov.	P30	JNP	Gyul	Pericarp	Decoction	25.00
			*Phellodendron amurense* Rupr.	P79	GNP	Hwangbyeongnamu	Bark, endodermis	Infusion, steep	76.92
			*Poncirus trifoliata* Raf.	P88	HNP, JNP	Taengjanamu	Fruit	Decoction, simmer	66.67
			*Zanthoxylum piperitum* (L.) DC.	P137	HNP, JNP	Chopinamu	Fruit	Decoction, oil	42.86
			*Zanthoxylum schinifolium* Siebold and Zucc.	P138	GNP, JNP	Sanchonamu	Fruit, seed	Oil	25.00
		Scrophulariaceae	*Paulownia coreana* Uyeki	P75	GNP	Odongnamu	Stem	Infusion	100.00
		Solanaceae	*Solanum nigrum* L.	P121	GNP, JNP	Kkamajung	Aerial part, fruit, leaf, root, whole part	Dried, infusion, juice	88.24
		Theaceae	*Camellia sinensis* L.	P22	JNP	Chanamu	Leaf	Decoction	100.00
		Ulmaceae	*Celtis sinensis* Pers.	P27	HNP	Paengnamu	Fruit	Dried, powder	100.00
			*Ulmus davidiana* var. *japonica* (Rehder) Nakai	P130	GNP	Neureumnamu	Bark	A sweet drink made from fermented rice, infusion	8.64

Intestinal disease	Plant	Asteraceae	*Cirsium japonicum* var. *spinossimum* Kitam.	P29	HNP	Gasieonggeongkwi	Root	Decoction	100.00
		Rosaceae	*Sorbus commixta* Hedl.	P123	JNP	Magamok	Fruit	Brewing	100.00

Stomach cramp	Animal	Phasianidae	*Gallus gallus domesticus* L.	A25	JNP	Dak	Whole part	Simmer	1.30
	Plant	Ranunculaceae	*Pulsatilla koreana* (Yabe ex Nakai) Nakai ex Nakai	P98	HNP	Halmikkot	Leaf	Rubbing	7.46

Stomach problem	Animal	Phasianidae	*Gallus gallus domesticus* L.	A25	JNP	Dak	Whole part	Simmer	1.30
		Asteraceae	*Atractylodes ovata* (Thunb.) DC.	P19	GNP	Sapju	Root	Powder	2.27
		Celastraceae	*Euonymus alatus* (Thunb.) Siebold	P43	GNP	Hwasallamu	Leaf, stem	Decoction, seasoned cooked vegetables	29.73
		Poaceae	*Oryza sativa* var*. terrestis* Makino	P73	HNP	Sandu	Seed	Porridge	7.69
	Plant	Rosaceae	*Potentilla chinensis* Ser.	P91	GNP	Ttakjikkot	Root	Infusion, raw	50.00
			*Prunus mume* Siebold and Zucc.	P94	GNP	Maesillamu	Fruit	Extraction	3.73
		Ulmaceae	*Ulmus davidiana *var*. japonica* (Rehder) Nakai	P130	GNP	Neureumnamu	Bark	Tea	1.85

Stomachic		Liliaceae	*Allium microdictyon* Prokh.	P10	JNP	Sanmaneul	Root	Decoction	50.00

Vomiting	Animal	Apidae	*Apis cerana* Fabricius	A7	GNP	Jaeraekkulbeol	Honey	Dissolution, raw	3.45
	Plant	Verbenaceae	*Vitex rotundifolia* L.f.	P134	HNP	Sunbiginamu	Fruit	Decoction	100.00

∗A: animal, P: plant, F: fungi, and AL: alga.

∗∗Region: JNP: Jirisan National Park, GNP: Gayasan National Park, and HNP: Hallasan National Park.

**Table 4:** Analytic results of ethnomedicinal practices recorded in the three national parks.

Results	JNP	GNP	HNP	Total
Species	166	76	58	220
Therapies (kinds)	272	176	92	490
Used parts	32	21	20	43
Preparations (modes)	28	34	21	42
Disorders (types)	20	11	16	24

**Table 5:** Number of times mentioned by informants and medicinal species for treating each disorder.

Diseases	JNP	GNP	HNP	Total
	Number of times mentioned (species)	Number of times mentioned (species)	Number of times mentioned (species)	Number of times mentioned (species)
Abdominal pain	161 (21)	516 (32)	82 (16)	759 (59)
Acute gastroenteritis	41 (8)		6 (3)	47 (11)
Constipation	115 (15)	142 (3)	36 (4)	293 (20)
Deficiency of intestinal function	19 (5)			19 (5)
Diarrhea	111 (36)	87 (8)	39 (9)	237 (48)
Dysentery	6 (4)	42 (4)	3 (1)	51 (8)
Enteritis	2 (1)			2 (1)
Enterotoxin			2 (2)	2 (2)
Gastralgia			5 (1)	5 (1)
Gastric cancer	14 (12)		3 (1)	17 (12)
Gastric ulcer	3 (3)	20 (2)		23 (3)
Gastritis	12 (3)	20 (5)	4 (1)	36 (9)
Gastroenteric trouble	238 (54)	755 (35)	118 (30)	1,111 (94)
Gastroptosis	3 (3)			3 (3)
Heartburn		28 (5)		28 (5)
Hema feces	6 (5)			6 (5)
Hematemesis	1 (1)			1 (1)
Hookworm	1 (1)		5 (1)	6 (2)
Indigestion	302 (52)	829 (31)	21 (7)	1,152 (72)
Intestinal ailment	2 (1)		1 (1)	3 (2)
Stomach cramp	1 (1)		5 (1)	6 (2)
Stomach problem	6 (4)	28 (5)	2 (1)	31 (7)
Stomachic	1 (1)			1 (1)
Vomiting	1 (1)	1 (1)	4 (1)	5 (2)

Total	1,040 (166)	2,468 (76)	336 (58)	3,844 (220)

**Table 6:** Informant consensus factor (ICF) of the communities of three national parks.

Disorders	JNP	GNP	HNP	Total
Abdominal pain	0.88	0.94	0.81	0.92
Acute gastroenteritis	0.83	−	0.60	0.78
Constipation	0.88	0.99	0.91	0.93
Deficiency of intestinal function	0.78	−	−	0.78
Diarrhea	0.68	0.92	0.79	0.80
Dysentery	0.40	0.93	1.00	0.86
Enteritis	1.00	−	−	1.00
Enterotoxin	−	−	+	+
Gastralgia	−	−	1.00	1.00
Gastric cancer	+	0.95	1.00	+
Gastric ulcer	+	−	−	0.91
Gastritis	0.82	0.79	1.00	0.77
Gastroenteric trouble	0.78	0.95	0.75	0.92
Gastroptosis	+	−	−	+
Heartburn	−	0.85	−	0.85
Hema feces	+	−	−	+
Hematemesis	+	−	−	+
Hookworm	+	−	1.00	0.80
Indigestion	0.83	0.96	0.70	0.94
Intestinal ailment	1.00	−	+	0.50
Stomach cramp	+	−	1.00	0.80
Stomach problem	0.40	0.85	1.00	0.80
Stomachic	+	−	−	+
Vomiting	−	+	1.00	0.75

−: Ailments were not mentioned in each national park.

+: Below 0.40.
